# Arabinogalactan proteins: focus on carbohydrate active enzymes

**DOI:** 10.3389/fpls.2014.00198

**Published:** 2014-06-11

**Authors:** Eva Knoch, Adiphol Dilokpimol, Naomi Geshi

**Affiliations:** Department of Plant and Environmental Sciences, University of CopenhagenCopenhagen, Denmark

**Keywords:** arabinogalactan proteins, type II arabinogalactan, plant cell wall, carbohydrate active enzymes, glycosyltransferase, glycoside hydrolase

## Abstract

Arabinogalactan proteins (AGPs) are a highly diverse class of cell surface proteoglycans that are commonly found in most plant species. AGPs play important roles in many cellular processes during plant development, such as reproduction, cell proliferation, pattern formation and growth, and in plant-microbe interaction. However, little is known about the molecular mechanisms of their function. Numerous studies using monoclonal antibodies that recognize different AGP glycan epitopes have shown the appearance of a slightly altered AGP glycan in a specific stage of development in plant cells. Therefore, it is anticipated that the biosynthesis and degradation of AGP glycan is tightly regulated during development. Until recently, however, little was known about the enzymes involved in the metabolism of AGP glycans. In this review, we summarize recent discoveries of carbohydrate active enzymes (CAZy; http://www.cazy.org/) involved in the biosynthesis and degradation of AGP glycans, and we discuss the biological role of these enzymes in plant development.

## Introduction

Arabinogalactan proteins (AGPs) are a family of proteoglycans found on the plasma membrane and in the cell walls of diverse species of plants. AGPs are synthesized by several post-translational modifications of proteins in the secretory pathway. The proteins generally contain repetitive dipeptide motifs, e.g., Ala-Pro, Ser-Pro, Thr-Pro, and Val-Pro, which are distinguished from the sequence motifs for extensin type glycosylation [e.g., Ser-(Pro)_2−3_] known as another major class of *O*-glycosylation in plants (Kieliszewski, 2001). The Pro residues are hydroxylated by prolyl 4-hydroxylases and further *O*-glycosylated by glycosyltransferases (GTs). Moreover, many AGPs are attached by a glycosylphosphatidylinositol anchor, which attaches AGPs to the plasma membrane, but can be cleaved by phospholipases (Wang, 2001; Schultz et al., 2004). AGPs on the plasma membrane and cell wall may also be processed by proteolytic activities and glycosyl hydrolases or transported by endocytotic multivesicular bodies to the vacuole where they are degraded (Herman and Lamb, 1992).

**Figure 1:**
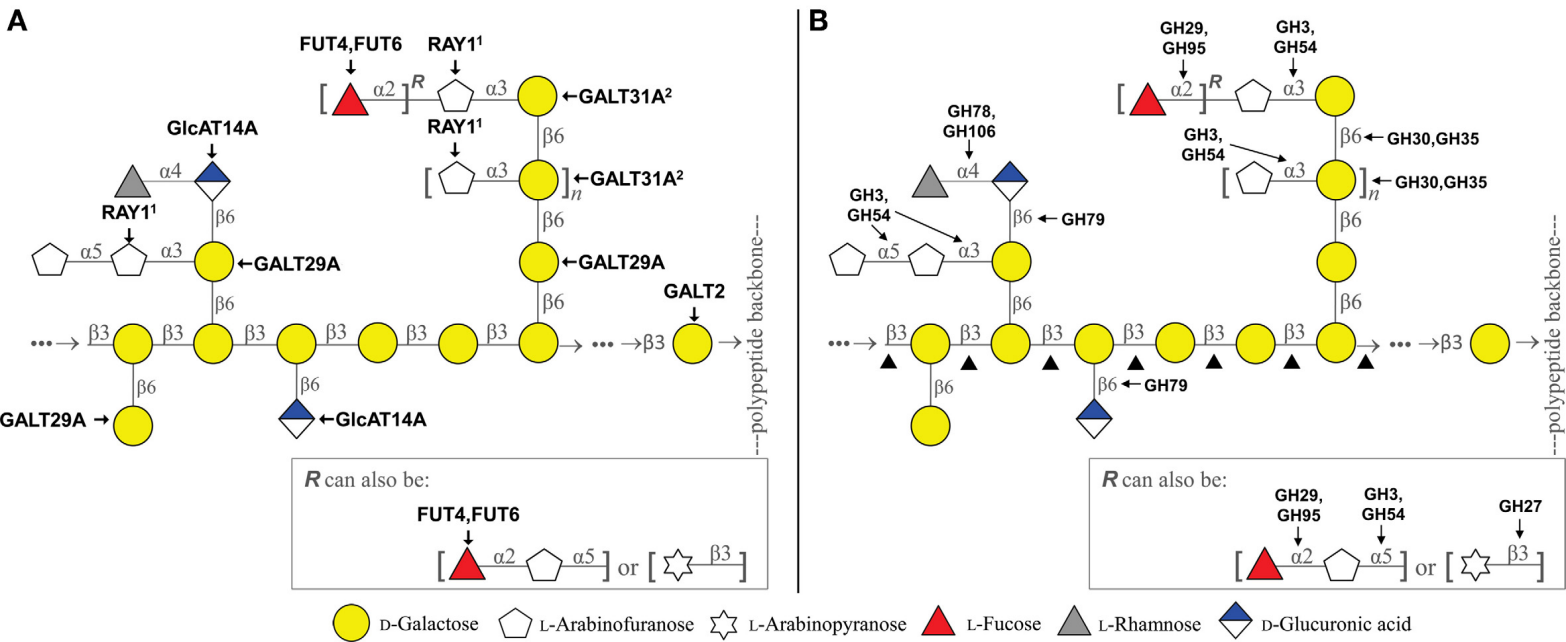
**Schematic representation of the type II arabinogalactan and the CAZy enzymes putatively involved in its biosynthesis (A) and degradation (B)**. ^1^Mutant analysis showed reduction of 3-linked Ara*f*, but activity of heterologously expressed protein in *N. benthamiana* showed β-linked product (Gille et al., 2013). ^2^Works cooperatively with AtGALT29A by forming a protein complex (Dilokpimol et al., 2014). ▲ includes GH16, GH35, GH43. The arabinogalactan model was modified from Tan et al. (2010) and Tryfona et al. (2012).

The glycan moiety of AGPs accounts for more than 90% of their total mass, which has been suggested to play an essential role in the function of AGPs, based on studies using synthetic phenylglycoside dyes (β-Yariv reagents) that specifically binds to the β-1,3-galactan moiety of AGPs (Kitazawa et al., 2013) as well as various monoclonal antibodies that recognize different AGP glycan epitopes (Seifert and Roberts, 2007). However, because of its complexity and heterogeneity, little is known about the structure-function relationship of AGP glycans. In fact, various structures have been reported for AGP glycans depending on samples and analytical methods. The common structural feature is a backbone of β-1,3-galactan, which is often substituted at *O*6 with side chains of β-1,6-galactan decorated further with arabinose, and less frequently also with fucose, rhamnose, and (methyl) glucuronic acid (Figures 1A,B). Tan et al. (2010) proposed that the backbone is composed of a repeat of a β-1,3-galactotriose unit with or without side chains, which is connected by β-1,6-linkages (kinks). This model is based on the AGPs synthesized onto synthetic peptides expressed in tobacco cells and analyzed by NMR (Tan et al., 2004, 2010). In this model, the side chains are rather short and composed of a single Gal decorated by 1–5 other sugars. However, longer β-1,6-galactan side chains have been reported for AGPs from radish root (Haque et al., 2005), wheat flour (Tryfona et al., 2010) and Arabidopsis leaf (Tryfona et al., 2012) based on the linkage and mass spectroscopy analysis.

Knowledge about each enzyme working on an individual step in the biosynthesis and degradation of AGP glycans is useful to understand the role of a particular sugar moiety of AGPs. This review outlines the recent findings for the carbohydrate active enzymes (CAZy; http://www.cazy.org/, Lombard et al., 2014) identified to be responsible for the biosynthesis and degradation of AGPs. The reader is referred to other excellent reviews for other topics with respect to structure, cell biological functions (Seifert and Roberts, 2007), localization, and commercial interests of AGPs (Nothnagel, 1997; Schultz et al., 1998; Majewska-sawka and Nothnagel, 2000; Gaspar et al., 2001; Showalter, 2001; Ellis et al., 2010; Tan et al., 2012).

## Glycosyltransferases involved in AGP biosynthesis

A large number of functionally distinct GTs are required for the biosynthesis of complex AGP glycans, e.g., β-1,3-galactosyltransferases (GalTs), β-1,6-GalTs, α-1,3- and α-1,5-arabinosyltransferases, fucosyltransferases, rhamnosyltransferases, glucuronosyltransferases, and glucuronic acid methyltransferases. Several GTs identified to date (Figure 1A and Table 1) are summarized below.

**Table 1:** **Characterized GTs and GHs, which process AGP-glycans**.

**CAZy Family**	**Activity**	**Protein name**	**Origin**	**No. of genes in Arabidopsis**	**Evidence^1^**	**Comments for enzyme activities and genetic manipulations**	**Selected references**
GT14	β-glucuronosyltransferase	AtGlcAT14A	*Arabidopsis thaliana*	11	HE-P, MA	GlcAT activity to β-1,3 and β-1,6-galactan; [GlcA]↓, [Gal]↑, [Ara]↓ in AG and enhanced cell elongation in seedlings in *atglcat14a*	Knoch et al., 2013
		AtGlcAT14B	*Arabidopsis thaliana*		HE-P	GlcAT activity to β-1,3 and β-1,6-galactan	Dilokpimol and Geshi, 2014
		AtGlcAT14C	*Arabidopsis thaliana*				
GT29	β-1,6-galactosyltransferase	AtGALT29A	*Arabidopsis thaliana*	3	HE-N	β-1,6-GalT activity to β-1,3 and β-1,6-galactan; interaction with AtGALT31A enhances the activity	Dilokpimol et al., 2014
GT31	Hydroxyproline O-galactosyltransferase	AtGALT2	*Arabidopsis thaliana*	33	HE-P, MA	GalT activity to hydroxylproline; [Yariv-precipitable AG]↓ in *galt2*; no detectable growth phenotype under normal growth condition	Basu et al., 2013
	β-1,6-galactosyltransferase	AtGALT31A	*Arabidopsis thaliana*		HE-N, -E; MA	β-1,6-GalT activity elongating β-1,6-galactan; mutant is embryo-lethal	Geshi et al., 2013
GT37	α-1,2-fucosyltransferase	AtFUT4	*Arabidopsis thaliana*	10	HE-B, MA	FucT activity to AGPs from BY2; [Fuc]↓ in AG in *fut4* and *fut6*; no [Fuc] in AG from *fut4/fut6*; no detectable growth phenotype under normal growth condition, but reduced root growth under salt stress	Wu et al., 2010; Liang et al., 2013; Tryfona et al., 2014
		AtFUT6	*Arabidopsis thaliana*				
GT77	Arabinofuranosyltransferase	AtRAY1^2^	*Arabidopsis thaliana*	19	HE-N, MA	β-ArafT activity to methyl β-Gal; [3-linked Ara]↓ in AG of *ray1*, slower root growth^2^	Gille et al., 2013
GH3	Exo-α-arabinofuranosidase	RsAraf1	*Raphanus sativus* L.	16^3^	HE-A	Cleaves α-linked Araf from AGPs, pectic α-1,5-arabinan, arabinoxylan. Overexpression in Arabidopsis resulted in [Ara]↓ in cell walls, but no growth phenotype	Kotake et al., 2006
GH16	Endo-β-1,3-galactanase	FvEn3GAL	*Flammulina velutipes*	33^4^	PUR, HE-P	Cleaves β-1,3-galactan in *endo* fashion	Kotake et al., 2011
GH27	Exo-β-arabinopyranosidase	SaArap27A	*Streptomyces avermitilis*	4	HE-S	Cleaves L-Ara*p* from *p*-nitrophenyl-β-L-Ara*p* and releases Ara from gum Arabic and larch AG	Fujimoto et al., 2009; Ichinose et al., 2009
GH30	Endo-β-1,6-galactanases	Tv6GAL	*Trichoderma viride*	0	HE-E	Cleaves β-1,6-galactan in endo fashion	Kotake et al., 2004
		FoGal1	*Fusarium oxysporum*		PUR, HE-E		Sakamoto et al., 2007
		Sa1,6Gal5A	*Streptomyces avermitilis*		HE-E		Ichinose et al., 2008
		Nc6GAL	*Neurospora crassa*		HE-P		Takata et al., 2010
GH35	Exo-β-1,3-,1,6-galactosidase	RsBGAL1	*Raphanus sativus*	18	PUR, HE-P	Exo activity to β-1,3- and β-1,6-Gal, but not to β-1,4-Gal. Cooperative degradation of AG with arabinofuranosidase and glucuronidase	Kotake et al., 2005
		AtBGAL4	*Arabidopsis thaliana*		HE-E, HE-I	Preferred cleavage at β-1,3 and β-1,4-linked Gal rather than β-1,6-linked Gal	Ahn et al., 2007
GH43	Exo-β-1,3-galactanase	Pc1,3Gal43A	*Phanerochaete chrysosporium*	2	HE-P	Cleaves β-1,3-linked Gal regardless the presence or absence of substituted side chains	Ishida et al., 2009
		Ct1,3Gal43A	*Clostridium thermocellum*		HE-E		Ichinose et al., 2006b
		Sa1,3Gal43A	*Streptomyces avermitilis*		HE-E		Ichinose et al., 2006a
		Il1,3Gal	*Irpex lacteus*		HE-P		Kotake et al., 2009
		SGalase1, 2	*Streptomyces* sp.		HE-E		Ling et al., 2012
GH54	Exo-α-arabinofuranosidase	NcAraf1	*Neurospora crassa*	0	HE-P	Broad specificity to α-1,3 and α-1,5-Araf, which includes AGPs, pectic arabinan, arabinoxylan	Takata et al., 2010
GH78	Exo-α-rhamnosidase	SaRha78A	*Streptomyces avermitilis*	0	PUR, HE-E	Releases Rha from gum Arabic	Ichinose et al., 2013
GH79	Exo-β-glucuronidase	AnGlcAase	*Aspergillus niger*	3	PUR, HE-P	Cleaves both GlcA and methyl GlcA from AG. Methyl GlcA from long β-1,6-galctan is cleaved, but not from short β-1,6-galactan	Haque et al., 2005; Konishi et al., 2008
		NcGlcAase	*Neurospora crassa*		HE-P	Cleaves GlcA from AG	Konishi et al., 2008
		AtGUS2	*Arabidopsis thaliana*		PUR, HE-A, MA	Cleaves *p*-nitrophenyl-β-D-GlcA. Mutant *atgus2*: [GlcA]↑, [Gal]↑, [Ara]↓, [Xyl]↓ in AG and reduced cell elongation in seedlings; overexpression AtGUS2: no [GlcA]; [Gal]↓, [Ara]↓, [Xyl]↑ in AG and enhanced cell elongation in seedlings	Eudes et al., 2008
GH95	Exo-α-1,2-fucosidase	AfcA	*Bifidobacterium bifidum*	1^5^	PUR, HE-E	Cleaves α-1,2-linked Fuc (linkage present in AG)	Nagae et al., 2007
GH106	Exo-α-rhamnosidase	Rham	*Sphingomonas paucimobilis*	0	PUR, HE-E	Broad specificity to α-Rha containing components. Involvement in AG degradation is unclear	Miyata et al., 2005

GTs are only from plants, while GHs are both from plants and microbial origins.

1 HE, activity demonstrated from heterologously expressed protein in: A, A. thaliana; B, tobacco BY2 cell; E, E. coli; N, N. benthamiana; P, P. pastoris; S, Streptomyces cinnamoneus, I, bacurovirus/insect cells; MA, mutant analysis.

2 Mutant analysis showed reduction of 3-linked Araf in AGPs, but heterologously expressed protein in N. benthamiana showed β-linked Ara to methyl β-Gal product, therefore a role of this protein in AGP glycosylation is not certain since arabinose exists as an α-linked sugar in AGPs (Gille et al., 2013).

3 Chracterized Arabidopsis enzymes demonstrated β-xylosidase activity toward xylan (Goujon et al., 2003; Minic et al., 2004).

4 Characterized Arabidopsis enzymes demonstrated xyloglucan endo-transferase activity (Rose, 2002).

5 Characterized Arabidopsis enzymes demonstrated α-1,2-fucosidase activity specifically toward xyloglucan (Léonard et al., 2008; Günl et al., 2011).

### β-galactosyltransferases

The first step in the glycosylation of AGPs is the transfer of Gal to hydroxyproline residues present in the peptide backbone. The Arabidopsis enzyme catalyzing this step was identified (At4g21060, AtGALT2, Basu et al., 2013). This enzyme belongs to the CAZy family GT31 and the recombinant protein expressed in *Pichia pastoris* demonstrated GalT activity transferring a Gal to hydroxyproline residues in the synthetic AGP peptides. Arabidopsis T-DNA knockout mutants contained reduced levels of Yariv precipitable AGPs and microsomes purified from mutants exhibited reduced levels of GalT activity compared to wild type. The mutant lines showed no detectable growth phenotype under normal growth conditions. Since the GalT activity was not completely abolished in the mutant microsomes, redundant activities encoded by other genes most likely exist. Nevertheless, based on this study, the quantity of AGP-glycans appears to not be crucial for plant development.

Another Arabidopsis GT from family GT31 encoded by *At1g32930* was also characterized. Recombinant enzyme expressed in *Escherichia coli* and *Nicotiana benthamiana* demonstrated β-1,6-GalT activity elongating β-1,6-galactan side chains of AGP glycans in *in vitro* assays (AtGALT31A; Geshi et al., 2013). *AtGALT31A* is expressed specifically in the suspensor cells of the embryo proper and T-DNA insertion lines showed abnormal cell division in the hypophysis and arrested further development of embryos. Therefore, functional AtGALT31A is essential for normal plant embryogenesis. How *AtGALT31A* that is expressed in suspensor cells influences cell division in hypophysis remains unknown.

*AtGALT29A* (*At1g08280*) was identified as a gene co-expressed with *AtGALT31A*. Recombinant enzyme expressed in *Nicotiana benthamiana* demonstrated β-1,6-GalT activities elongating β-1,6-galactan and forming 6-Gal branches on β-1,3-galactan of AGP glycans (Dilokpimol et al., 2014). Moreover, Förster resonance energy transfer analysis revealed an interaction between AtGALT29A and AtGALT31A when both proteins are expressed as C-terminal fluorescent fusion proteins in *Nicotiana benthamiana* (Dilokpimol et al., 2014). The protein complex containing heterologously expressed AtGALT29A and AtGALT31A were purified and demonstrated increased levels of β-1,6-GalT activities by the AtGALT29A single enzyme. These results suggest cooperative action between AtGALT31A and AtGALT29A by forming an enzyme complex, which could be an important regulatory mechanism for producing β-1,6-galactan side chains of type II AG during plant development.

### β-glucuronosyltransferase

An Arabidopsis GT from family GT14 encoded by *At5g39990* was identified as a glucuronosyltransferase involved in the biosynthesis of AGP glycans (AtGlcAT14A; Knoch et al., 2013). The enzyme expressed in *Pichia pastoris* demonstrated β-GlcAT activity by adding GlcA to both β-1,6- and β-1,3-galactan. Arabidopsis possesses 11 proteins in the GT14 family, of which two additional proteins encoded by *At5g15050* and *At2g37585* also demonstrated the same β-GlcAT activities and were named AtGlcAT14B and AtGlcAT14C, respectively (Dilokpimol and Geshi, 2014). The T-DNA insertion lines contained reduced levels of GlcA substitution of β-1,6-galactobiose and β-1,3-galactan in their AGPs compared to wild type. In addition to the altered levels of GlcA, a marked increase of Gal and a decrease of Ara were detected in the mutant AGPs. Mutant lines showed an increased cell elongation rate in dark grown hypocotyls and light grown roots during seedling growth compared to wild type. Since several sugars were altered in the mutant AGPs lines, it is unlikely that the observed phenotype is solely a consequence of the reduced levels of GlcA, but most likely related to the dynamic conformational changes of AGP glycans caused in the mutants.

### α-fucosyltransferase

Two Arabidopsis GTs from family GT37 were identified as α-1,2-fucosyltransferases involved in the biosynthesis of AGP glycans (AtFUT4 and AtFUT6, encoded by *At2g15390* and *At1g14080*, respectively; Wu et al., 2010). AGPs from tobacco BY2 cells contain no fucose, but heterologous expression of AtFUT4 and AtFUT6 in BY2 cells resulted in fucosylated AGPs. The recombinant enzymes purified from BY2 cells demonstrated fucosyltransferase activity to endogenous AGPs. Single Arabidopsis T-DNA insertion lines, *atfut4* and *atfut6*, contained reduced levels of fucose in AGPs, and the double T-DNA insertion line *fut4/fut6* contained no detectable fucose in its AGPs (Liang et al., 2013); however, no obvious phenotype was observed in both types of mutants when they were grown under normal conditions. Differences between wild type and mutants were only seen in seedlings grown under salt stressed condition, where the mutant lines showed reduced root growth compared to wild type (Liang et al., 2013; Tryfona et al., 2014). This was somewhat surprising because the Arabidopsis *mur1* mutant, which is defective in a GDP-mannose-4,6-dehydratase (Bonin et al., 1997), contained a 40% reduction of fucose in root extracts (Reiter et al., 1997) and showed a 50% reduction of cell elongation rate in roots (Van Hengel and Roberts, 2002). The decrease of root cell elongation in *mur1* was previously attributed to the lack of fucose in AGPs (Van Hengel and Roberts, 2002), but the findings by Liang et al. (2013) and Basu et al. (2013) refute that hypothesis. The molecular changes behind the *mur1* phenotype are hard to pinpoint. Mutants affected in *N*-glycan fucosylation show reduced root growth under salt stress conditions (Kaulfürst-Soboll et al., 2011), and *mur2* plants, which lack only xyloglucan fucosylation, have no visible root phenotype (Van Hengel and Roberts, 2002; Vanzin et al., 2002). The *mur1* phenotype might be due to under-fucosylated rhamnogalaturonan II, or to the combination of several cell wall polysaccharides deficient in fucose.

### α-arabinofuranosyltransferase

An Arabidopsis GT encoded by *At1g70630* (named REDUCED ARABINOSE YARIV1, RAY1; Gille et al., 2013), was characterized as a putative arabinofuranosyltransferase since the mutation caused a reduced level of arabinofuranose (Ara*f*) in its AGPs. This GT belongs to the GT77 family, which also contains *XEG113*, the mutation of which results in the reduction of β-linked arabinose in extensin (Gille et al., 2009). Therefore, Ara*f* transferase activity that makes β-linkages was expected for RAY1, and indeed, microsomes isolated from *Nicotiana benthamiana* after expression of recombinant RAY1 demonstrated β-Araf transferase activity to methyl β-Gal. The T-DNA insertion lines contained reduced levels of 3-linked Ara in its AGP fractions compared to AGPs from wild type, and the mutant plants exhibited slower root growth as well as a reduced rosette size and inflorescence. However, β-1,3-Linked Ara*f* has not been reported in AGPs, therefore the involvement of RAY1 in the biosynthesis of AGP glycans remains unclear.

## Glycoside hydrolases

Glycoside hydrolases (GHs) acting on AGPs are potentially very important for the metabolism of these glycoproteins. AGPs from tobacco stylar transmitting tissue are degraded as the pollen tube grows and the released sugars are considered to be used as the carbohydrate resource necessary for the elongation of pollen tubes (Cheung et al., 1995). Similarly, rapid turnover of AGPs are observed in suspension cell culture and millet seedlings (Gibeaut and Carpita, 1991) and a substantial amount of AGPs are considered to be hydrolyzed to free sugars and recycled in the cytosol for the synthesis of new glycans (Gibeaut and Carpita, 1991) or degraded in the vacuole (Herman and Lamb, 1992). The appearance of distinct AGP epitopes in a developmentally regulated manner might be controlled by GHs in the cell walls. Additionally, the occurrence of free AG glycans detached from proteins observed in the cell walls may be a result of GH actions.

For the hydrolysis of AGP glycans, several GHs are required, e.g., β-galactosidases, β-galactanases, α-arabinofuranosidases, β-arabinopyranosidases, β-glucuronidases, α-fucosidases, and α-rhamnosidases. AGP degrading GHs from microbial origin have been relatively well characterized, while only a few plant GHs have been reported to degrade AGP glycans. Below is an overview for those GHs reported to possess hydrolase activity of AGP glycans from both microbial and plant origins (Figure 1B and Table 1).

### β-galactosidase and β-galactanase

A GH16 from the fungus *Flammulina velutipes* was characterized as an endo-β-1,3-galactanase degrading the AGP glycan β-1,3-galactan backbone (Kotake et al., 2011). The enzyme activity is distinct from other GH16 enzymes, which comprise β-1,3- and β-1,3:1,4-glucanases, xyloglucan endo-transglycosylase and β-agarase activities. Arabidopsis contains 33 proteins in the GH16 family and they are characterized as xyloglucan endo-transglycosylases and their homologs (Kaewthai et al., 2013).

Several enzymes of microbial origin in GH30 have been characterized as endo-β-1,6-galactanases that hydrolyze β-1,6-galactan side chains of AGP glycans (Kotake et al., 2004; Sakamoto et al., 2007; Ichinose et al., 2008; Takata et al., 2010). These β-1,6-galactanases were originally categorized as part of the GH5 family, but were moved to the GH30 family after additional bioinformatic analysis by (St John et al., 2010). Tv6GAL from *Trichoderma viride* was the first β-1,6-galactanase cloned and characterized (Kotake et al., 2004). This enzyme specifically recognizes β-1,6-galactan of AGPs and releases galactose and β-1,6-linked galactooligomers with a degree of polymerization from two to five. Efficiency of the hydrolysis of β-1,6-galactan is increased by pretreatment of the AGP substrate with α-L-arabinofuranosidase. The β-1,6-galactanases from *Fusarium oxysporum* (FoGAL1, Sakamoto et al., 2007), *Streptomyces avermitilis* (Sa1,6Gal5A, Ichinose et al., 2008), and *Neurospora crassa* (Nc6GAL, Takata et al., 2010) act in a similar manner, releasing galactose and β-1,6-galactobiose from β-1,6-galactan of AGP. All three enzymes show increased activity on de-arabinosylated AGP, similarly to Tv5GAL. Plants do not have any proteins classified in the GH16 CAZy family.

Several microbial β-1,3-galactosidases from GH43 have been characterized as exo-β-1,3-galactanase that degrades the β-1,3-galactan backbone of AGP glycans (Ichinose et al., 2005, 2006a,b; Kotake et al., 2009). All of these enzymes show similar substrate specificity and degrade β-1,3-linked Gal regardless of the substitution of side chains, which results in free Gal from unsubstituted β-1,3-galactan and side chains attached to β-1,3-linked Gal. Therefore, the GH43 enzymes have been used to release side chains from AGP glycans for structural analysis (Tryfona et al., 2010, 2012; Geshi et al., 2013; Knoch et al., 2013). The GH43 CAZy family contains two uncharacterized Arabidopsis proteins.

In plants, β-1,3-galactosidase has been purified from radish (*Raphanus sativus*) seed extracts (Kotake et al., 2005). Based on the deduced protein sequence, the enzyme RsBGAL1 was classified to the GH35 CAZy family. This enzyme was expressed heterologously in *Pichia pastoris* and demonstrated GH activity by degrading β-1,3- and β-1,6-galactan in an exo manner, but not β-1,4-galactan. The efficiency of degradation of AGP glycans by RsBGAL1 alone was limited, but co-treatment with arabinofuranosidase and glucuronidase resulted in the release of up to 90% of the bound sugars from AGPs, indicating the synergy of those GHs in the degradation of AGP glycans.

### α-arabinofuranosidase and β-arabinopyranosidase

An α-arabinofuranosidase from the fungus *Neurospora crassa* with a broad substrate specificity toward AGPs, pectic arabinan, and arabinoxylan was identified and classified to the CAZy GH54 family (NcAraf1, Takata et al., 2010). This enzyme was heterologously expressed in *Pichia pastoris* and demonstrated α-arabinofuranosidase activity on both α-1,3- and α-1,5-linked Ara*f*. NcAraf1 has been used extensively for the structural characterization of AGP glycans together with galactosidases, galactanases, and glucuronidases (Tsumuraya et al., 1990; Okemoto et al., 2003; Kotake et al., 2004, 2009; Konishi et al., 2008; Tryfona et al., 2010, 2012). Arabidopsis does not have proteins in the GH54 family.

Plant α-arabinofuranosidase acting on AGP glycans is classified to family GH3. Kotake et al. (2006) purified an α-arabinofuranosidase from radish seeds and named it RsAraf1. The recombinant enzyme expressed in Arabidopsis demonstrated hydrolytic activity on radish AGPs, pectic α-1,5-arabinan and arabinoxylan. Transgenic Arabidopsis overexpressing RsAraf1 showed decreased levels of Ara in the cell wall, but no obvious growth phenotype was observed compared to wild type plants.

β-Arabinopyranose generally represents only a minor part of Ara in AGP glycans, but has been reported from acacia, larch and wheat flour AGPs (Aspinall et al., 1958; Groman et al., 1994; Odonmazig et al., 1994; Tryfona et al., 2010). β-Arabinopyranosidase has been identified from *Streptomyces avermitilis* (Ichinose et al., 2009). The enzyme, named SaArap27A, belongs to family GH27, and the recombinant enzyme expressed in *Streptomyces* demonstrated the release of L-arabinopyranoside (Ara*p*) from p-nitrophenyl-β-L-arabinopyranoside, as well as the release of L-arabinose from gum Arabic and larch AG. Arabidopsis contains four uncharacterized proteins in the GH27 family.

### β-glucuronidase

Microbial β-glucuronidases are found in family GH79. Two fungal GH79 β-glucuronidases from *Neospora crassa* (NcGlcAase) and *Aspergillus niger* (AnGlcAase) have been cloned and recombinant proteins expressed in *Pichia pastoris* demonstrated β-glucuronidase activity (Konishi et al., 2008). AnGlcAase and NcGlcAase share high homology in their amino acid sequences, but possess slightly different substrate specificity. Both enzymes recognize unsubstituted and 4-methyl substituted β-GlcA on AGPs, but AnGlcAase cleaves both GlcA and 4-methyl GlcA with an equal efficiency, while NcGlcAase preferably cleaves GlcA and only small amounts of 4-methylGlcA. Arabidopsis contains three proteins in the GH79 family.

In plants, β-glucuronidases (GUS) are ubiquitously present and their activity is associated with cell elongation (Sudan et al., 2006). Eudes et al. (2008) partially purified a GUS from Arabidopsis stems, which cleaves *p*-nitrophenyl-β-D-glucuronide. The corresponding gene was identified and classified to the GH79 family (AtGUS2, At5g07830, Eudes et al., 2008). The T-DNA knockout insertion lines exhibited increased levels of GlcA, whereas plants overexpressing AtGUS2 lacked detectable levels of GlcA in their AGP fractions. The T-DNA insertion mutant of *AtGUS2* showed no clear changes in the elongation rate of plant organs, whereas the overexpression lines exhibited increased elongation of roots and stems. The increase of cell elongation observed in the overexpression lines of *AtGUS2* resembles similar observations of the *atglcat14a* T-DNA insertion lines. Although reduced levels of GlcA is observed in both types of plants, the altered profiles of other sugars present in AGP glycans are inconsistent. Therefore, it is unlikely that the increase of cell elongation is solely caused by the reduction of GlcA levels in AGPs.

### α-fucosidase

α-Fucosidases from various prokaryotic and eukaryotic sources have been characterized, and several of them are commercially available. α-Fucosidases are classified into two GH families: GH29 and GH95 (Lombard et al., 2014). α-Fucosidases from GH29 are capable of hydrolyzing various types of linkages, mainly α-1,3/1,4-linked Fuc, whereas GH95 enzymes are active solely on α-1,2-linked Fuc. Only one GH from Arabidopsis is found in each of these GH families. *At2g28100* (AtFUC1; Zeleny et al., 2006) belongs to GH29 and the recombinant enzyme expressed in *Pichia pastoris* demonstrated α-1,3/1,4-fucosidase activity (Zeleny et al., 2006). *At4g34260* (Fuc95A, AXY8; Léonard et al., 2008; Günl et al., 2011) belongs to GH95 and the enzyme heterologously expressed in *Nicotiana benthamiana* demonstrated α-1,2-fucosidase activity (Léonard et al., 2008). α-1,2-Fuc is present in both xyloglucan and AGP glycans, but Fuc95A (AXY8) acts specifically on α-1,2-Fuc on xyloglucan and not on AGPs (Günl et al., 2011). α-1,2-Fucosidase purified from *Xanthomonas manihotis* apparently cleaves α-1,2-fucose on AGP glycans and has been used for the product analysis of AGP fucosyltransferases (AtFUT4 and AtFUT6, Wu et al., 2010). Wu et al. (2010) also used α-1,3/4-fucosidase from almond meal for the characterization of the Fuc linkage. Both enzymes are commercially available, but are not classified to CAZy GH families.

### α-rhamnosidase

Microbial α-rhamnosidases are classified into three GH families: GH28, GH78, and GH106 (Fujimoto et al., 2013; Lombard et al., 2014). An α-rhamnosidase from *Aspergillus niger* in GH28 was identified as specifically degrading pectic rhamnogalacturonan (RgxB; Martens-Uzunova et al., 2006). Several α-rhamnosidases from GH78 have been characterized and recently an α-rhamnosidase from *Streptomyces avermitilis* expressed in *Escherichia coli* demonstrated an α-rhamnosidase that releases rhamnose (Rha) from gum Arabic AGPs (SaRha78A; Fujimoto et al., 2013; Ichinose et al., 2013). GH106 exclusively contains bacterial α-rhamnosidases, of which only one has been characterized to date. This α-rhamnosidase was purified from *Sphingomonas paucimobilis* FP2001 (Rham; Miyata et al., 2005) and the enzyme expressed in *Escherichia coli* demonstrated α-rhamnosidase activity on a broad range of substrates containing α-Rha. One of those substrates is α-rhamnosyl-1,4-galactose, but whether the enzyme hydrolyzes α-rhamnosyl-1,4-GlcA, which is found as part of the side chains of AGP glycans, remains unknown. Arabidopsis contains 28 proteins in the GH28 family, but no plant proteins are present in GH78 and GH106. Among Arabidopsis GH28s, only pectin polygalacturonase has been characterized (Torki et al., 2000; Markovic and Janecek, 2001). Functions of other plant GHs in GH28 remain unknown.

## Conclusions

Microbial GHs working on the degradation of plant AGPs have been reported in several studies, but little was known about the enzymes working on the biosynthesis and degradation of AGPs in plants. Recent discovery of plant GTs/GHs working on AGPs, together with the technical development of in-depth structural analysis of complex AGP glycans, has broadened our knowledge for AGP metabolism significantly. On the other hand, the attempt to elucidate the biological role of each sugar moiety or a particular part of AGP glycan structure by investigating knockout mutants or overexpressors of those enzymes did not result in straightforward answers. For instance, a mutation in *AtGlcAT14A* did not result in a sole reduction of GlcA but also exhibited an increase of Gal and a reduction of Ara in the AGP glycan as well as enhanced cell elongation in seedlings. Furthermore, the overexpression of *AtGUS2* resulted in a reduction of GlcA, Gal and Ara in AGP glycans and seedlings showed increased cell elongation, similarly to *atglcat14a*. The developmentally regulated appearance of different AGP glycan epitopes is well known, but the results available thus far are inconclusive concerning the molecular role of a particular part of AGP glycans in plant growth and development.

The carbohydrate active enzymes involved in the AGP metabolism have just begun to be identified and characterized. Further investigation of the remaining members in the AGP glycosylation pathway and their role *in vivo* is needed to understand the role of CAZy enzymes in relation to AGP glycans, the cell wall architecture, and in plant growth and development.

### Conflict of interest statement

The Review Editor, Dr. Ulvskov, declares that, despite being affiliated to the same institution and having collaborated with author(s) Eva Knoch, Adiphol Dilokpimol, Naomi Geshi, the review process was handled objectively. The authors declare that the research was conducted in the absence of any commercial or financial relationships that could be construed as a potential conflict of interest.
